# Supplementary material for: Reactive oxygen species in endothelial signaling in COVID-19: Protective role of the novel peptide PIP-2
Source: PLoS One. 2024 May 21;19(5):e0289854. doi: 10.1371/journal.pone.0289854 (PMC11108150; doi:10.1371/journal.pone.0289854)
Supplement: Attachment — Submitted filename: RESPONSE TO REVIEWERS.docx [file pone.0289854.s002.docx]

**RESPONSE TO REVIEWERS:**

We thank the editor, the editorial staff, and the reviewers for their support. The reviewer requested a minor change.

*Reviewer #2:*

*In immunofluorescence figures, some photos are not the same size or do not align well. No further comments*

Our response: We have aligned the images and have brought all images except Figure 7, where the inset is enlarged to a bigger size for visual clarity.
